# Colloidal fibers and rings by cooperative assembly

**DOI:** 10.1038/s41467-019-11915-1

**Published:** 2019-09-02

**Authors:** Joon Suk Oh, Sangmin Lee, Sharon C. Glotzer, Gi-Ra Yi, David J. Pine

**Affiliations:** 1 Center for Soft Matter Research, Department of Physics, New York University, New York, NY 10003 USA; 2 Department of Chemical Engineering, University of Michigan, Ann Arbor, MI 48109 USA; 3 Department of Materials Science and Engineering, University of Michigan, Ann Arbor, MI 48109 USA; 4 Biointerfaces Institute, University of Michigan, Ann Arbor, MI 48109 USA; 5 Department of Chemical Engineering, Sungkyunkwan University, Suwon, 16419 Republic of Korea; 6 Department of Chemical and Biomolecular Engineering, New York University, Brooklyn, NY 11201 USA

**Keywords:** Colloids, Colloids, Self-assembly

## Abstract

Janus colloids with one attractive patch on an otherwise repulsive particle surface serve as model systems to explore structure formation of particles with chemically heterogeneous surfaces such as proteins. While there are numerous computer studies, there are few experimental realizations due to a lack of means to produce such colloids with a well-controlled variable Janus balance. Here, we report a simple scalable method to precisely vary the Janus balance over a wide range and selectively functionalize one patch with DNA. We observe, via experiment and simulation, the dynamic formation of diverse superstructures: colloidal micelles, chains, or bilayers, depending on the Janus balance. Flexible dimer chains form through cooperative polymerization while trimer chains form by a two-stage process, first by cooperative polymerization into disordered aggregates followed by condensation into more ordered stiff trimer chains. Introducing substrate binding through depletion catalyzes dimer chains to form nonequilibrium rings that otherwise do not form.

## Abstract (web summary)

Janus colloids with an attractive patch on the surface are model systems to explore structure formation but experimental realizations of such particles are rare. Here, the authors report a scalable method to precisely vary the Janus balance over a wide range and observe the formation of various structures including fibers, bilayers, and nonequilibrium rings catalyzed by substrate binding.

## Introduction

The self-assembly of proteins into crystals or biologically active structures is a complex dynamical process that depends critically on both the shape and the chemical heterogeneity (patchiness) of the protein surface^1,2^. Colloidal particles have served as model systems for exploring the role of shape in self-assembly^3–5^, and notably for lock-and-key interactions using dimpled colloids^6^.

The role of chemical heterogeneity may play an even greater role than shape in self-assembly, but there are few experiments on model systems where the strength and extent of the chemical heterogeneity are well controlled^7^. One of the simplest systems exhibiting complex structures due to chemical heterogeneity is the self-assembly of Janus colloids with one attractive patch, with the rest of the particle being repulsive. The different structures they form depend on the interaction strength *ε* of the attractive patch and the Janus balance, defined as the fractional area *χ* of the attractive patch. Taken together, these play the same role as the hydrophilic-lipophilic balance (HLB) for surfactants. Although Janus particles with various patch ratios and interaction ranges have been explored in computer simulations^8–14^, systematic experimental studies are rare, as it has proven difficult to adjust the patch ratio of Janus particles using available fabrication methods^15^. Previous experimental work with hydrophobic patches on otherwise hydrophilic Janus particles suggests that kinetic trapping can select nonequilibrium structures over equilibrium ones, so the ability of structures to anneal and equilibrate can play a role in structure formation.

Here, we introduce a versatile and scalable method to synthesize Janus particles with well-controlled Janus balance *χ*. One face of the Janus particles is selectively functionalized with DNA having self-complementary sticky ends. We use DNA-mediated attraction rather than a hydrophobic attraction for three reasons: (1) the DNA-mediated interaction is thermally-reversible, (2) properly-functionalized DNA-coated colloids can anneal after they bind to each other^16^, and (3) the strength of the DNA-mediated attraction can be continuously varied from essentially zero to about 10 *k*_*B*_*T* by varying the temperature over a range of about 10 °C^17,18^.

Because the self-assembly of micron-sized particles can be observed in real time under an optical microscope, which is not possible for proteins, we can follow their assembly. In addition, we conduct molecular dynamics (MD) simulations, which reproduce the formation process. Taken together, our experimental and computational observations reveal the dynamical processes that lead to the formation of colloidal chains at different patch ratios, as well as a new dynamical process of surface-catalyzed ring formation of dimer chains^19–21^.

## Results

### Fabrication of Janus particles

**Fig. 1:**
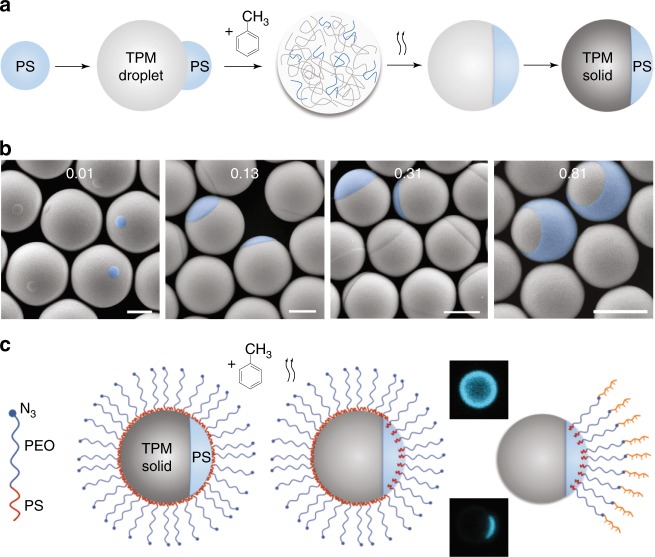
Janus particle fabrication. **a** Left to right: A reactive silicone oil droplet, 3-(trimethoxysilyl)propyl methacrylate (TPM), is heterogeneously grown on the surface of a polystyrene (PS) microsphere by hydrolysis and condensation. Toluene is then added to swell the TPM-grown PS particles, forming fully liquid emulsion droplets in which PS and TPM oligomers are homogenously dissolved. Next, the PS and TPM oligomer droplets phase-separate as toluene gradually evaporates, resulting in a biphasic colloid composed of a PS cap and a TPM oligomer body. Finally, the TPM oligomer body is highly cross-linked by radical polymerization resulting in monodisperse Janus particles composed of solid PS and TPM patches (PS-TPM particles). **b** SEM images show PS-TPM Janus particles with various patch ratios ranging from 0.01 to 0.81. False color aids identification of the PS patches. Scale bar = 1 μm. **c** Amphiphilic block copolymers, consisting of a hydrophobic polystyrene (PS) block and a hydrophilic poly(ethylene oxide) (PEO) block with an azide functional end-group (PS-*b*-PEO-N_3_), adsorbs to the entire surface of the PS-TPM particle (PS-*b*-PEO-N_3_ are not drawn to scale: PS-*b*-PEO-N_3_ block is ~10 nm thick; particles are ~1000 nm in diameter). Adding toluene leads to selective swelling of the PS patch, as the PS patch is not crosslinked, while the TPM is highly crosslinked. The PS block of the PS-*b*-PEO-N_3_ to penetrates the swollen PS patch. After deswelling, the PS block is trapped inside the PS patch while the PEO block remains outside. DBCO-functionalized DNA (DBCO-DNA) is coupled to the azide functional group at the end of the PEO brush using SPAAC^44^. The block copolymer coupled with DNA on the TPM surface is removed by washing with a surfactant solution (Triton X-100, 1-wt%). The fluorescence images show the DNA-coated Janus particles before (top) and after (bottom) washing, confirming that only the PS patch is coated with DNA, which is fluorescently labeled

**Fig. 2:**
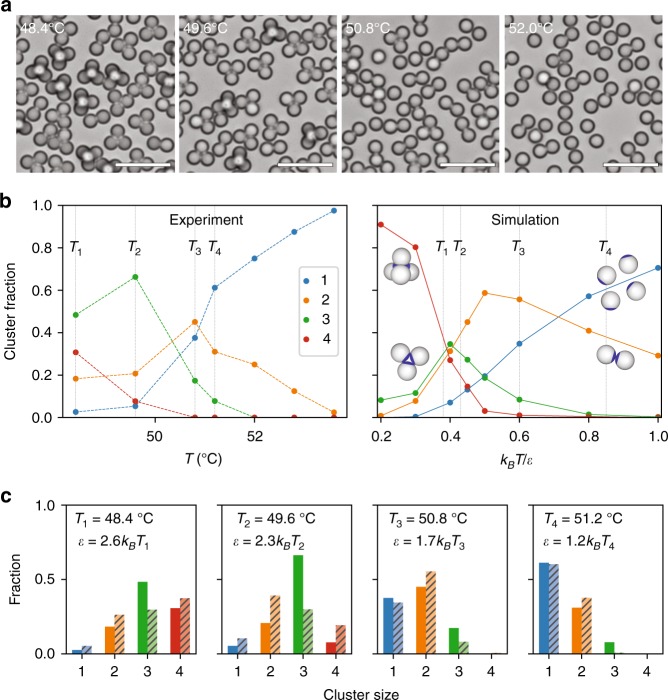
Assembly of Janus particles with patch ratio of *χ* = 0.13. **a** Bright-field images show self-assembled clusters of Janus particles at various temperatures. Particles disassemble at high temperature and self-assemble at low temperature at the DNA-coated surfaces, forming singlets and clusters of dimers, trimers, and tetramers. Scale bar = 10 μm. **b** Distribution of singlets and clusters of 2, 3, and 4 particles at different temperatures from experiment (left) and computer simulation (right). Singlets and dimers are dominant at high temperature while trimers and tetramers are dominant at low temperature. **c** Cluster fractions as a function of temperature from experiment (solid color bars) and computer simulation (hashed). The depth *ε* of the attractive effective potential due to DNA binding, indicated in each panel, is determined by choosing the values of *k*_*B*_*T*/*ε* in the simulations that best match the experimental distributions of cluster fractions for the different temperatures indicated in (**b**). Low temperature favors the formation of larger clusters, which are limited by geometry to four particles for the patch ratio *χ* = 0.13 shown here

The Janus particles, we make consist of a polystyrene (PS) patch and a cross-linked 3-(trimethoxysilyl)propyl methacrylate (TPM) patch^22^. We introduce a fabrication method that combines incompatible elements, PS and TPM, in a cosolvent emulsion droplet and then uses phase separation to create the patches. The technique, which is scalable, is described in Fig. 1a. The Janus balance *χ* can be readily controlled from 0.01 to 0.81 by varying the amount of TPM monomer introduced during synthesis relative to the size of the PS particle, as illustrated in Fig. 1b and in Supplementary Figs. 1 and 2. The PS surface of PS-TPM particles is selectively functionalized with a di-block copolymer brush using a swelling-deswelling process^23^. The copolymer brush is terminated with DNA sticky ends by strain-promoted alkyne-azide cycloaddition (SPAAC)^24^ (Fig. 1c and Supplementary Fig. 1). The DNA density on the particles is 1 DNA strand per 10.2 nm^2^, or 3.2 nm between polymer grafts. The DNA-coated PS-TPM particles reversibly associate and dissociate upon thermal cycling (Fig. 2a and Supplementary Movie 1).

### Self-assembly of particles with small patch ratios

First, we present the results for the self-assembly of Janus particles with a patch ratio of *χ* = 0.13. The bright-field and fluorescent images in Fig. 2a and Supplementary Fig. 3 show the cluster formation of dimers, trimers, and tetramers. Geometrically, the minimum patch ratio for a tetramer is *χ* = 0.092. No pentamers or higher-order clusters are observed, as these are geometrically prevented by the small size of the patches. To form these clusters, we heated the sample above the melting temperature *T*_*m*_ = 53 °C. Cooling the sample to 52.0 °C, just below *T*_*m*_, allows the clusters to anneal and equilibrate. At 52.0 °C, only singlets and dimers are observed. As the temperature is decreased, trimers form at 50.8 °C and then increase in number at 49.6 °C. Finally, at 48.4 °C, the fraction of trimers decreases as the fraction of tetramers increases, as shown in Fig. 2a.

The relative populations of singlets, dimers, trimers, and tetramers were measured as a function of temperature and are shown in the left panel of Fig. 2b. These are compared to the relative populations obtained from simulations, shown in the right panel of Fig. 2b, which exhibit the same general trend with the system tending towards trimers and tetramers at low temperature and towards singlets and doublets at higher temperature. The simulations show that to achieve the observed relative changes in the cluster fractions, the ratio *k*_*B*_T/*ε* must change by more than a factor of 2, from about 0.38 to 0.85. In the experiments, the temperature changes by only a few degrees around a characteristic temperature of 325 K, which means that changes in the interaction strength *ε* = *ε*(*T*) must account for the observed changes in cluster populations. In Fig. 2c, we show histograms of the cluster fractions measured in experiments and simulation at four different temperatures, which are indicated by the gray vertical lines in each panel in Fig. 2b. The four values of *k*_*B*_T/*ε* in the simulations are chosen to best match the experimental distributions for the four different temperatures. From this, we see that *ε* changes from 1.2 *k*_*B*_*T* at 51.2 °C to 2.6 *k*_*B*_*T* at 48.4 °C, a change that is consistent with the expected temperature dependence of the DNA-mediated attractive interaction^17,25^.

### Cooperative chain formation

**Fig. 3:**
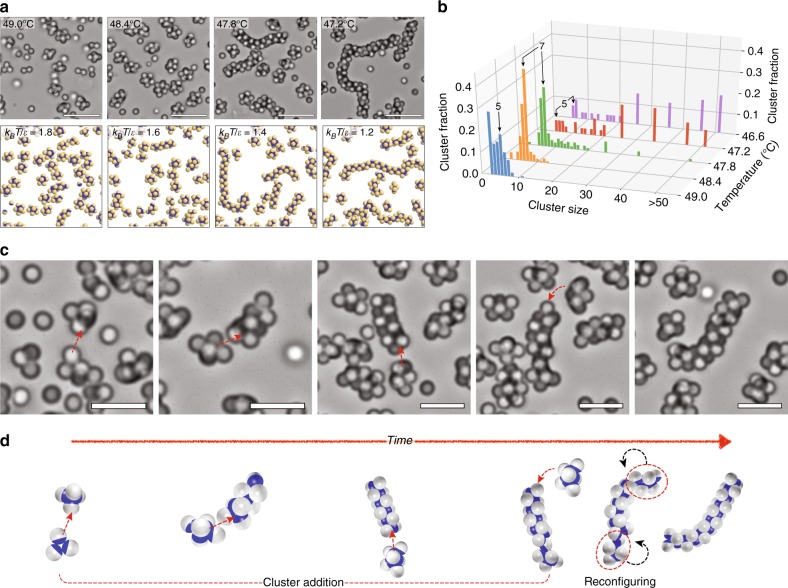
Self-assembly of Janus particles to clusters and chains for a patch ratio of 0.3. **a** Bright-field images above and simulation renderings below show self-assembled structures of Janus particles at different temperatures. Particles self-assemble into small clusters at higher temperature and longer chain-like structures at lower temperature, where the DNA-mediated attraction between patches is greater. **b** Fraction of clusters in clusters of different sizes as a function of temperature. At higher temperatures, small clusters with 8 or fewer particles dominate. As the temperature is lowered, chains develop and grow longer, mostly by the addition of small clusters but occasionally by fusion of long chains. For cluster sizes greater than 20, the histogram bars integrate over ten or more bins: 21–30, 31–40, 41–50, and >50. **c** Snapshots and **d** illustrations show the formation of chain structures by collective polymerization through cluster addition and reconfiguration

DNA-coated PS-TPM particles with larger patch ratios *χ* can form more than three bonds per particle, which leads to larger structures. Figure 3 shows the general trend of self-assembly for particles with patch ratios *χ* from 0.30 to 0.41. Above the melting temperature *T*_*m*_ = 50 °C, particles are well dispersed and diffuse freely. When the temperature decreases to 49 °C, the particles start to bind to each other forming clusters of two to seven particles. When the temperature further decreases to around 48 °C, we observe mixtures of clusters and short chains (see also Supplementary Movie 2). At 47.2 °C, long chain structures, some of them with branches, are formed as the number of clusters decreases. This picture is consistent with simulation results, which show the same trends as the strength of the attractive interaction *ε*(*T*) increases (Supplementary Movie 3 and Supplementary Fig. 5b). The formation of chains occurs exclusively by cluster-cluster binding, most frequently by the addition of clusters of 4–7 particles to longer chains, but occasionally by longer chains fusing. Figure 2c and Supplementary Fig. 6 show the sequential addition of small clusters to a growing chain, which is typical for the growth of dimer chains (Supplementary Movie 4).

This chain formation process is known as cooperative assembly^26^, where a chain grows by the addition of clusters rather than by the addition of individual particles^15^. A key characteristic of cooperative assembly is the coexistence of long chains in equilibrium with small clusters. Indeed, the equilibrium distribution of aggregate sizes, shown in Fig. 3b and Supplementary Fig. 7, shows that small clusters coexist with chains. Upon closer examination, we see that at 49.0 °C, there are only clusters of 8 particles or fewer, with a small excess of pentamers. When the temperature is lowered to 48.4 °C, the distribution becomes strongly peaked around 7 particles, with a few chains appearing with a dozen particles or more. Upon lowering the temperature to 47.8 °C, the number of small clusters (notably sizes of 6 and 7) decreases, and the number of chains grows. This trend continues as the temperature is decreased, with the growth of successively longer chains while a small but finite fraction of small clusters, with no fewer than 5 particles, always remains.

**Fig. 4:**
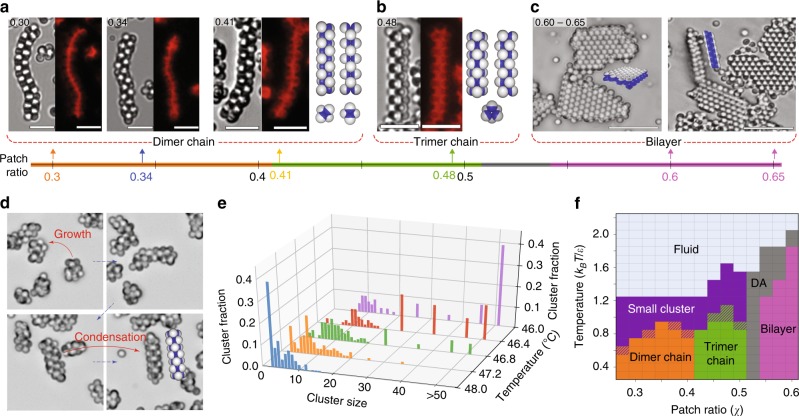
DNA-mediated self-assembly of Janus particles with patch ratios from 0.3 to 0.65. **a** Bright-field and fluorescent images for self-assembled structures of DNA-coated Janus particles with patch ratios from 0.3 to 0.65. Particles with patch ratios from 0.3 to 0.41 self-assemble into flexible dimer chains. The colors on the patch-ratio axis follow those of the phase diagram shown in frame (**f**). **b** Particles with a patch ratio of 0.48 self-assemble into stiff densely-packed trimer chains. **c** Particles with patch ratios of 0.6 and 0.65 self-assemble into bilayer structures. The right-most image shows a cross-section of a bilayer structure. **d** Still frames from Supplementary Movie 5 showing the assembly of a trimer chain. Disordered chains form and grow by combining with clusters, and subsequently condense into trimer chains. **e** Fraction of clusters that are found in clusters of different size. For cluster sizes greater than 20, the histogram bars integrate over ten or more bins: 21–30, 31–40, 41–50, >50. **f** Phase diagram as a function of patch ratio and temperature, obtained by computer simulation (DA: disordered aggregates)

The microstructure of the chains that are formed from particles with *χ* = 0.30–0.41 is one of alternating dimers, as illustrated in Figs. 3d, 4a, and Supplementary Fig. 5f. Each dimer, which is oriented perpendicular to the contour of the chain, serves as a rotational axis for chain bending. Adjacent dimers along the chain have rotation axes oriented 90° with respect to each other, rendering dimer chains very flexible, which is observed in the Supplementary Movies 2–4. Each particle in a dimer-chain binds to five other particles.

By contrast, when *χ* = 0.48, chains consisting of an alternating stack of triangular trimers form, as shown in Fig. 4b and Supplementary Fig. 5c, g. These chains are much stiffer than the dimer chains. Like the dimer chains, however, they form from small clusters and grow by cooperative polymerization, but in this case by a two-stage process, which is very similar to the two-stage process described in simulations of one-patch colloids by Vissers et al.^27^. The chains that initially form are disordered and flexible, and remain so for some time after formation, after which they transform into stiff ordered trimer chains (Supplementary Fig. 8). The transition from disordered to ordered chain is driven by the increased number of bonds that can be formed when the chain orders^27^. The process can be observed in real time under a microscope (Supplementary Movie 5) and in simulation (Supplementary Movie 6). Illustrative still frames from Supplementary Movie 5 are shown in Fig. 4d.

Figure 4e and Supplementary Fig. 9 show the equilibrium distribution of trimer chains measured at different temperatures. As for the case of dimer chains, long trimer chains coexist with a population of smaller clusters. In this case, however, the distribution of smaller clusters is broader and more disordered than it is for dimer chains. It is also striking how rapidly the population of long trimer chains increases as the temperature decreases. By decreasing the temperature from 48 °C to 46 °C, the system goes from having only small aggregates of 10 particles or fewer to a situation where roughly 40% of the clusters consist of 50 or more particles. This behavior is consistent with the phase diagram obtained from simulations and shown in Fig. 4f, namely that small clusters form at the higher temperatures for *χ* < 0.55 while various chains form at the lower temperatures (see also Fig. 3a).

The structure of an alternating stack of triangles is consistent with our simulations and with those of Luitjen^15^, but in contrast to the experimental observation of Boerdijk-Coxeter (BC) helices of Chen et al. for gold-capped hydrophobic-hydrophilic Janus particles with *χ* *∼* 1/2, which are less stable than trimer chains based on free energy calculations^15^. Chen et al. suggested that the BC helices, which also grow by the addition of small clusters, are kinetically trapped and thus do not achieve the equilibrium trimer chain structure. Our chains, however, do reach the predicted equilibrium structure. Recently, Fejer et al. pointed out that the thickness of the gold caps in the experiments of Chen et al. is greater at the center of the caps than at the edges, which leads to an orientation-dependent van der Waals interaction between the gold caps^9^. The analysis of Fejer et al. suggests that orientation-dependent interaction between gold caps favors BC helices. Thus, the BC structures may be equilibrium structures after all rather than kinetically-trapped states.

### Bilayers

When the patch ratios are increased to 0.60 and 0.65, the Janus particles self-assemble into bilayers, as shown in Fig. 4c (Supplementary Movie 7 and Supplementary Fig. 10), which agrees with our simulations and those of others (Supplementary Movie 8, Supplementary Figs. 5d, h and 11)^10,12^. Initially, particles form amorphous aggregates that gradually rearrange into hexagonally-packed bilayers upon annealing. Once they form bilayers, growth occurs at the edges of pre-existing bilayers. Top- and side-views of bilayers in Fig. 4c confirm that the particles assemble into two layers of an ordered hexagonal array. Figure 4f shows the phase diagram determined from our simulations for Janus particles as a function of the dimensionless temperature *k*_*B*_*T/ε* vs. patch ratio *χ*. The phase diagram is consistent with our experiments showing the same progression of structures occurring at patch ratios consistent with our experiments.

### Surface-catalyzed formation of rings

One of our most striking observations is the formation of substrate-catalyzed rings of dimer chains when a weak attractive depletion interaction is introduced. It is well known that polymer micelles can induce a depletion attraction between particles^28,29^ and between particles and substrates^30^. We empirically selected 1.0% w/w of polymeric surfactants (F127 from BASF Corp.), above its critical micelle concentration (<<0.1% w/w around our annealing temperatures), as this introduces a weak depletion attraction that avoids depletion-mediated bulk crystallization and leaves the DNA-mediated attractive interaction as the primary force driving self-assembly.

**Fig. 5:**
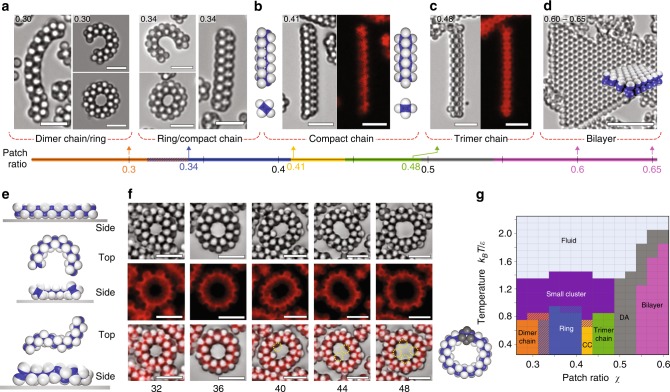
DNA-mediated self-assembly of Janus particles in the presence of depletion force. **a**–**c** Optical microscope images for self-assembled structures of DNA-coated Janus particles with various patch ratios between *χ* = 0.3 and 0.65 in the presence of 1% of F127, which forms micelles and introduces a weak attractive depletion interaction. **a** When *χ* = 0.3, particles self-assemble into dimer chains as well as ring structures, which form only when there is a depletion interaction. **b** For *χ* = 0.34, the same structures are observed, as well as densely-packed chains which we call compact chains (CC). For *χ* = 0.41, most chains are compact chains. **c** However, the structures of trimer chains (*χ* *=* 0.48) and **d** bilayers (*χ* *=* 0.6 and 0.65) are unchanged. **e** Half of the particles of a dimer chain can touch the substrate if the chain is straight (top) or has a constant radius of curvature (middle). However, when the dimer chain curvature is not constant, as for an S-shaped chain (bottom), one or more particles in the chain detach from a flat substrate. **f** Bright field (top) and fluorescent (middle) optical microscope images of rings consisting of 32–48 particles. Yellow dashed circles in bright field/fluorescent overlay (bottom) show the locations octahedral defects that appear for *χ* = 0.34. Illustration on the right shows the structure of a single octahedral defect. **g** Phase diagram of Janus particles obtained by simulation in the presence of a depletion interaction (*ε*_*dep*_ = 0.1)

For a patch ratio of *χ* = 0.30, dimer chains are observed, just as for the case without depletion. The most significant effect of adding a depletion interaction is that it pushes dimer chains against the walls of the sample container, as the depletion attraction between spheres and a flat substrate is twice as strong as between spheres^30^. Geometrically, exactly half of the particles in a perfectly straight dimer chain can touch and adhere to a flat substrate via the depletion interaction (Fig. 5e and Supplementary Fig. 12). Doing so maximizes the depletion attraction and lowers the free energy of the system. When a dimer chain bends, the geometrical constraints of a dimer chain dictate that the number of particles that can touch the substrate must decrease unless the radius of curvature of the chain is constant over the entire length of the chain, as illustrated in Fig. 5e. Thus, a fluctuating dimer chain minimizes its free energy by adopting a circular C-shape of constant curvature when interacting with a flat substrate under the influence of the depletion interaction. This is exactly what we observe in both experiments and simulations, as illustrated in Fig. 5a (see also Supplementary Figs. 13, 14, and Supplementary Movie 9). The strength of the depletion attraction is difficult to determine experimentally, but rings form in the simulations when the strength of the depletion attraction is 5% that of the DNA. The constraint of constant curvature leads to the formation of closed rings, also shown in Fig. 5a and Supplementary Movie 10, which are stable indefinitely. By contrast, rings are never observed in the absence of a depletion interaction. This reflects the entropic barrier to ring formation in the bulk suspension. However, restricting conformations to 2D structures with constant curvature reduces entropy to the point that the energetic advantage of forming rings overcomes the entropic barrier and thus catalyzes ring formation. Once the chain overcomes the barrier, rings persist even in the absence of the depletion effect (Supplementary Fig. 15).

We observe rings of varying size and number of particles. Geometrically, the smallest ring that can form without spheres overlapping consists of 32 particles, which is indeed what we observe. Larger rings may form, with the constraint that the total number of particles be divisible by four, as the dimers must come in pairs so the rings can close. We observe rings of 32, 36, 40, 44, and 48 particles in both experiment and simulation, which is consistent with the geometrical reasoning.

Rings are also observed for Janus particles with *χ* = 0.34. Rings of 32 and 36 particles form without defects. However, defects generally appear in larger rings with 40, 44, and 48 particles. The defects are localized and consist of 6 particles in the chain adopting an octahedral conformation, as illustrated by the schematic of a ring with a single defect in Fig. 5f. Such defects are stiff, which reduces the flexibility of the chain and thus reduces its conformational entropy. At the same time, each defect results in an additional interparticle bond, which lowers the energy of the chain, compensating for the entropic cost. The octahedral defects proliferate in the larger rings, which are more flexible and have more conformational entropy than the smaller rings. The defects are highlighted in the rings with 40, 44, and 48 particles in Fig. 5f.

These octahedral defects can proliferate along a chain for patch ratios from 0.34 to 0.41 to the point that the chain consists of almost nothing but octahedral defects, effectively creating a new type of chain, which we call a compact chain (Fig. 5b and Supplementary Fig. 16). We also observe the same depletion-induced transition in our simulations (Supplementary Fig. 17). For *χ* = 0.34, dimer chains coexist with compact chains, often with a few dimer-chain defects, which make flexible kinks between the stiff compact chain segments (Fig. 5a and Supplementary Movie 11). Bright field and fluorescent images of compact chains for *χ* = 0.34 and 0.41 are shown in Fig. 5a, b along with illustrations of the compact chain. In fact, the situation is subtler than this. Just as dimer chains can exist with a few stiff octahedral defects, so too can compact chains exist with a few flexible dimer-chain defects. These dimer defects reveal themselves as kinks in otherwise stiff compact chains. Supplementary Movie 11 shows a compact chain with a dimer-defect kink that heals and then reappears further down the chain.

Figure 5g shows the phase diagram obtained from our simulations for the case where there is a weak depletion interaction and a flat surface. Once again, it is consistent with our experiments showing the same progression of phases with transitions between phases. Note that, in the region of the ring phase, our experiments show rings coexisting with dimer and compact chains because not every chain can overcome the kinetic and energetic barrier to transform into the rings within the 24-h duration of experiments.

## Discussion

Despite their simplicity, colloidal spheres with tunable Janus balance exhibit some of the most basic features in cooperative self-assembly, which is also thought to be active in biological molecules like proteins, although the evidence is indirect^31^. From our observations, we can draw several lessons.

Small aggregates are self-limiting if the Janus balance is small but can serve to nucleate and grow fibers when the Janus balance reaches about 0.3. Thus, a small increase in the Janus balance can lead to the growth of long fibers.

Fiber growth generally occurs by cooperative polymerization, where fibers grow not by adding single particles but by adding small particle aggregates.

The chain structure is sensitive to the Janus balance, with different types of fibers forming when the Janus balance changes from about 0.4 to 0.47. Moreover, the dynamical pathways for fiber formation depend sensitively on the Janus balance. The slow two-step process by which trimer chains grow when the Janus balance is about 0.47 becomes a rapid, single-step process for dimer chains when the Janus balance falls to about 0.4.

We emphasize that all the structures formed by our DNA-coated Janus particles are equilibrium structures. This follows from the ability of the DNA-coatings we use to thermally anneal, as discussed and demonstrated in previous publications^16,24^. This is in contrast to the nonequilibrium structures reported by Kang et al.^32,33^ and possibly to those reported by Chen et al^15^.

Finally, we find that introducing a depletion interaction, which is the colloidal analogue of molecular solvation interactions, can have profound effects. First, it can alter the structure of the fibers that form, as illustrated by the formation of stiff compact chains instead of flexible dimer chains when *χ* ∼1/3. Second, it can catalyze entirely new and unanticipated structures. The dimer-chain rings we observe illustrate how interaction with a substrate can impose constraints on a polymer that favor certain configurations that otherwise would be very unlikely. This is reminiscent of ring-like amyloid protein aggregates, which can adopt different conformations when bound to a substrate^1^, and may have high toxicity due to their topology^34^. By using colloids, we can follow the dynamics of the structural evolution. DNA-coated Janus colloids thus provide a simple but effective model of fiber formation and interactions with substrates, which underscores their utility as a model system for exploring the dynamics of structure formation at a level that remains difficult to achieve with biological macromolecules.

## Methods

### Fabrication of PS-TPM Janus particles

One hundred microliters of carboxylate polystyrene microspheres (CPS, diameter = 0.7 μm, 4% w/v, from Thermo Fisher Scientific) is dispersed in 2 ml of NH_3_ solution (0.3 vol%). 30–300 μl of 3-(trimethoxysilyl)propyl methacrylate (TPM) is then added to the suspension. The suspension is shaken in an orbital shaker (IKA MS 3 basic) at a speed of 500 rpm. After one hour, 2 ml of NH_3_ solution is added to the suspension followed by an additional two hours of rotation, which condenses TPM droplets onto the CPS. 100 μl of toluene is then added and vigorously vortexed to swell the PS particles and TPM oligomer droplets with toluene. Then, 0.5 ml of sodium dodecyl sulfate solution (5% w/v) and 0.5 ml of potassium carbonate solution (100 mM) are added to the suspension. Toluene is evaporated at 80 °C with gentle stirring for an hour. 0.01 g of 2,2′-azobis(2-methylpropionitrile) is added to the suspension followed by vigorous vortexing. The suspension is then stored in an oven at 70 °C for more than 12 hours to solidify the TPM. After polymerization, the PS-TPM particles are washed with an EtOH/DI water mixture (30% EtOH) two times and with DI water two times. The PS-TPM Janus particles are dispersed in DI water at a concentration of around 10%. The monodispersity of the Janus particles is confirmed by SEM analysis. The size variation is around 3%. The PS Janus patch area and internal morphology of the Janus particles are determined by surface tension between phases as predicted using a Surface Evolver^35^, which is confirmed experimentally by selective removal of the PS or TPM sections (Supplementary Fig. 2).

### Functionalization of PS-*b*-PEO with azide functional groups

0.1 g of polystyrene-b-polyethylene oxide diblock copolymer (PS-b-PEO, M_w_ = 10,300 g/mol, PS = 3800 g/mol, and PEO = 6500 g/mol, from Polymer Source) is dissolved in 2 ml of anhydrous dichloromethane. 42 μl of triethylamine (0.3 mmol) and 23.5 μl of methanesulfonyl chloride (0.3 mmol) are added to the polymer solution. The mesylation reaction is carried out for 24 hours at room temperature. After the reaction, the solvent is removed by vacuum evaporation. MeOH containing 3 vol% of 37% HCl is poured into the mesylated PS-b-PEO (PS-b-PEO-Ms) to dissolve the remaining reagents. The solution is stored at subzero temperature for several hours and the phase-separation of PS-b-PEO-Ms proceeds. The supernatant containing impurity is removed using centrifugation. This process is repeated twice with pure MeOH and then PS-b-PEO-Ms is fully dried under vacuum. The PS-b-PEO-Ms is dissolved in 2 ml of anhydrous dimethylformamide containing 0.01 g of sodium azide (0.15 mmol). The solution is vigorously stirred at 65 °C for 24 h to functionalize with azide groups. An excess of diethyl ether is poured into the solution to precipitate the azide-functionalized PS-b-PEO (PS-b-PEO-N_3_) and separated by centrifugation. PS-b-PEO-N_3_ is redissolved in MeOH and precipitated using diethyl ether two times for purification. PS-b-PEO-N_3_ is fully dried under vacuum. 1 mM of PS-b-PEO-N_3_ aqueous solution (solution is milky due to the micellization of block copolymer) is prepared and stored in a refrigerator.

### DNA-functionalization of PS-TPM patch particles

Twenty microliters of suspension of PS-TPM patchy particles (1 wt%), 40 μl of PS-b-PEO-N_3_ (1 mM), 180 μl of DI water, and 160 μl of EtOH are mixed in a microcentrifuge tube. 2 μl of toluene is added to the suspension and shaken for an hour to swell the PS patch with toluene. Toluene is slowly then evaporated at room temperature by shaking the suspension with the cap open for an hour. Then around 1.1 ml of DI water is added to the mixture. After the deswelling process, the Janus particles coated with PS-b-PEO-N_3_ are washed with DI water several times and redispersed in 190 μl of PBS buffer (salt concentration is adjusted with NaCl to 400 mM) containing Pluronic F127 (0.05 wt%). 10 μl of dibenzocyclooctyne-modified single-stranded DNA (DBCO-DNA, 70 μM) is added to the suspension and the coupling reaction is conducted for 24 h. After the coupling reaction, the DNA-coated Janus particles are washed with 1% Triton X-100 solution to remove the DNA-coupled brushes adsorbed on the TPM surface and washed with DI water several times and stored in TE buffer. The DNA sequences are as follows: DBCO-Cy5-(T)_16_GCGC (self-complementary, from Integrated DNA Technologies). The fluorescent images of the same assemblies in Supplementary Fig. 3 confirm that the particles are connected to each other only through DNA-coated PS patches.

### Assembly of DNA-coated Janus particles

Capillary tubes are plasma-treated and functionalized with hexamethyldisilazane vapor to make the surface hydrophobic. The hydrophobic tube is filled with 1% F127 solution to coat the inner surface with the polymeric surfactant. The excess F127 solution is removed by blowing with nitrogen gas. DNA-coated Janus particles are dispersed in PBS buffer with F127 (0.05% or 1%). The suspension is loaded into the functionalized capillary tube and both ends of the tube are sealed with UV-curable resins. The samples are loaded on a temperature gradient, annealed for several hours to a day and directly observed under a microscope.

### Characterization

The morphology of the Janus particles is characterized using a field-emission scanning electron microscope (Merlin, Carl Zeiss). Bright field and fluorescent images are taken using an inverted microscope (Eclipse Ti, Nikon) microscope. For the cluster size distribution, we counted around 330 Janus particles for *χ* = 0.13 and 1000 Janus particles for *χ* = 0.3 and 0.47 from bright-field images.

### Measurement of DNA density on the Janus particles

DNA-coated Janus particles, where the DNA contains an internal Cy5 dye, are dispersed in PBS buffer. The suspension is loaded to a flow cytometer (BD LSRII, BD Biosciences) to measure the fluorescent intensity of the single particles. A standard calibration curve is achieved by measuring standard particles (Quantum™ Cy™5 MESF, from Bangs Laboratories) on the same day. The DNA density per Janus patch is determined by comparing the fluorescent intensity of each sample to the calibration curve.

### Molecular dynamics

Molecular dynamics (MD) simulations are performed to simulate the self-assembly behavior of the DNA-coated Janus particles. The MD method implemented in the HOOMD-blue simulation package^36,37^ is available as open source, which can be downloaded at http://glotzerlab.engin.umich.edu/hoomd-blue/. HOOMD-blue runs both on central processing units (CPUs) and graphics processing units (GPUs) and is optimized for parallel computing with multiple CPUs and GPUs using MPI domain decomposition. Most simulations of the present work were run on Intel Xeon E5–2680v3 CPUs and NVIDIA Tesla K20X GPUs.

A minimal model is developed to simulate a large number of Janus particles in reasonable timescale. The Weeks-Chandler-Andersen (WCA) potential^38^ is applied on whole sphere to create a rigid core, and the Morse pair potential^39^ is applied on the patch region to reproduce the self-complementary DNA hybridization (Supplementary Fig. 4a, b). Note that, we used the Morse pair potential in this study because it is convenient to control the range of attraction, not physical reason. The depletion effect caused by surfactants is modeled in implicit way by applying the Lennard-Jones (LJ) pair potential^40^ on the Janus particles.

The MD simulations were performed with *N* = 1000 particles for most self-assembly systems, which is large enough size to form multiple superstructures which are usually composed of dozens of particles, but small enough to form those structures in a reasonable timescale. Typically, 48 GPU hours are taken for 10^8^ MD timesteps (dt = 0.005) with Brownian dynamics for each state point. We used periodic boundary conditions in two dimensions and one non-periodic boundary for creating a bottom where particles sediment and assemble. Gravitational force (*F*_*G*_) is applied toward the bottom, which is converted from the experimental gravitational height ($k_BT/F_G \approx$ 0.3 µm)^41^ of the present work (Supplementary Fig. 4c). Packing density ($\phi$) is kept constant as 0.1 for all system, which is approximately calculated by $N \times v_0/V_{assemble}$, where N and ***v***_0_ are the number of particles and volume of a particle, respectively. The volume of the assembly space (*V*_*assemble*_) is calculated by multiplying an assembled height ($\sim \! 2\sigma$) and two other simulation box lengths, where $\sigma$ is the unit length of simulation and the diameter of a Janus particle.

### Simulation model of DNA-coated Janus particles

DNA hybridization between patches is modeled by Morse potential (Supplementary Fig. 4a),

1 $$V_{\mathrm{P}} = D_0[\exp \left( { - 2\alpha \left( {r - r_0} \right)} \right) - 2\exp \left( { - 2\alpha \left( {r - r_0} \right)} \right)]$$

where *D*_0_ and $\alpha$ is depth and width of the potential well with the unit of $\varepsilon$ and $1/\sigma$, respectively, and *r*_0_ is position of the potential minimum with $\sigma$ unit. The ratio ($\chi$) of DNA-patch is angularly modulated^42^ as follow:

2 $$V_{{\mathrm{AM}}\_{\mathrm{P}}} = \frac{{V_{\mathrm{P}}}}{{(1 + \exp \left( { - w\left( {\hat n_i \cdot \hat r_{ij} - \chi } \right)} \right))(1 + \exp \left( { - w\left( {\hat n_j \cdot \hat r_{ji} - \chi } \right)} \right))}}$$

where $\hat n_i$ is unit vector from particle *i* center to its patch center, $\hat r_{ij}$ is the center-to-center unit vector of particle *i* and *j*, and *w* is the angular sharpness ranging from 0 to infinity. When *w* is infinity, this potential is identical to the Kern-Frenkel model^43^. The particle contacts via non-patch regions are modeled by the Weeks-Chandler-Andersen (WCA) potential^38^ (Supplementary Fig. 4b) to simulate volume exclusion:

3 $$V_{{\mathrm{NP}}}\left( r \right) = \left\{ {\begin{array}{*{20}{l}} {4\varepsilon _{{\mathrm{NP}}}\left[ {\left( {\frac{{\sigma _{{\mathrm{NP}}}}}{r}} \right)^{12} - \left( {\frac{{\sigma _{{\mathrm{NP}}}}}{r}} \right)^6} \right] - 4\varepsilon _{{\mathrm{NP}}}\left[ {\left( {\frac{{\sigma _{{\mathrm{NP}}}}}{{r_{{\mathrm{cut}}}}}} \right)^{12} - \left( {\frac{{\sigma _{{\mathrm{NP}}}}}{{r_{{\mathrm{cut}}}}}} \right)^6} \right],} \hfill & {r \, < \, r_{{\mathrm{cut}}}} \hfill \\ \hskip 25pt {0,} \hfill & {r \ge r_{{\mathrm{cut}}}} \hfill \end{array}} \right.$$

where $r_{{\mathrm{cut}}} = \sigma _{{\mathrm{NP}}} \times 2^{1/6}$. The depletion attraction (*V*_dep_) between monopatch particles induced by depletants is implicitly modeled^40^ via the Lennard-Jones potential:

$$V_{{\mathrm{dep}}}\left( r \right) = 4\varepsilon _{dep}\left[ {\left( {\frac{{\sigma _{{\mathrm{dep}}}}}{r}} \right)^{12} - \left( {\frac{{\sigma _{{\mathrm{dep}}}}}{r}} \right)^6} \right]$$

The $\varepsilon _{dep}$ can vary depending on the concentration of depletant. The combined effect of depletion attraction and patch or non-patch contacts is achieved by summation. The potential parameters are listed in the Table 1 below.

**Table 1:** Potential parameters for the MD simulation

Morse potential (*V*_P_)		WCA potential (*V*_NP_)		LJ potential (*V*_dep_)	
Parameter (unit)	Value	Parameter (unit)	Value	Parameter (unit)	Value
$D_0$ ($\varepsilon$)	5.0	$\varepsilon _{{\mathrm{NP}}}$ ($\varepsilon$)	1.0	$\varepsilon _{{\mathrm{dep}}}$ ($\varepsilon$)	0.0–0.14
$\alpha$ ($1/\sigma$)	7.0	$\sigma _{{\mathrm{NP}}}$ ($\sigma$)	1.0	$\sigma _{{\mathrm{dep}}}$ ($\sigma$)	1.0
$r_0$ ($\sigma$)	1.0				

Angular modulation parameter (*w*) is 20.0 for all system

### Simulation protocol

For the self-assembly simulations, each run follows the following steps: (i) Locate the Janus particles randomly in 3-D simulation box without overlapping between particles and give a random orientation to each particle. (ii) To create a gravitational effect, apply a constant force (*F*_*G*_) uniformly to the whole system toward a uniform direction. Note that the simulation box should be non-periodic along the *F*_*G*_ direction to sediment particles, but periodic to the two other directions. (iii) Running the system at constant temperature (T*) by checking the potential energy of the whole system in each $10^3\tau$ to make sure that the system is equilibrated. Typically, $10^8\tau$ was enough to equilibrate the system. Snapshots for each stage are shown in Supplementary Fig. 4c.

For investigating the depletion effect on the transformation of dimer chain into the ring and the compact chain, a simulation is initialized from an ideally constructed dimer chain (*N* = 36) and the depletion effect is applied to both particles and bottom. By varying strength of the depletion effect ($\varepsilon _{{\mathrm{dep}}}$ = 0–0.14, $\Delta \varepsilon _{{\mathrm{dep}}}$ = 0.01) and the patch ratio, we check the results observed within the $10^8\tau$ simulation time. The temperature (T*) is kept constant for each state point.

For the phase diagram (Figs. 4f and 5g in main figures), each superstructure (dimer chain, trimer chain and bilayer) is built and initialized from a low enough temperature ($T^ \ast \le 0.3$) to keep the structure stable. Then, we gradually increase the temperatures and obtained the melting temperature of each phase. In the presence of the depletion effect (Fig. 5g), the ring phase and the compact chain (CC) phase are determined when the initial dimer chain transform into the structure.

## Supplementary information

Supplementary Information

Description of Additional Supplementary Files

Supplementary Movie 1

Supplementary Movie 2

Supplementary Movie 3

Supplementary Movie 4

Supplementary Movie 5

Supplementary Movie 6

Supplementary Movie 7

Supplementary Movie 8

Supplementary Movie 9

Supplementary Movie 10

Supplementary Movie 11

## Data Availability

The datasets generated during and/or analyzed during the current study are available from the corresponding authors upon reasonable request.

## Code availability

The HOOMD-Blue software package used in the study is available online (http://glotzerlab.engin.umich.edu/hoomd-blue/).
