# Supplementary material for: Colloidal fibers and rings by cooperative assembly
Source: Nat Commun. 2019 Sep 2;10:3936. doi: 10.1038/s41467-019-11915-1 (PMC6718632; doi:10.1038/s41467-019-11915-1)
Supplement: Supplementary file 1 — Supplementary Information [file 41467_2019_11915_MOESM1_ESM.pdf]

Supplementary Information for

Colloidal fibers and rings by cooperative assembly

Joon Suk Oh[1], Sangmin Lee[2], Sharon C. Glotzer[2,3,4*], Gi-Ra Yi[1,5*], David J. Pine[1,5,6*]

[1]*Center for Soft Matter Research, Department of Physics, New York University, New York, NY 10003, USA*
[2]*Department of Chemical Engineering,* [3]*Department of Materials Science and Engineering,* [4]*Biointerfaces Institute, University of Michigan, Ann Arbor, MI 48109, USA.*
[5]*Department of Chemical Engineering, Sungkyunkwan University, Suwon, 16419, Republic of Korea*
[6]*Department of Chemical and Biomolecular Engineering, New York University, Brooklyn, NY, 11201, USA*

Supplementary Figures

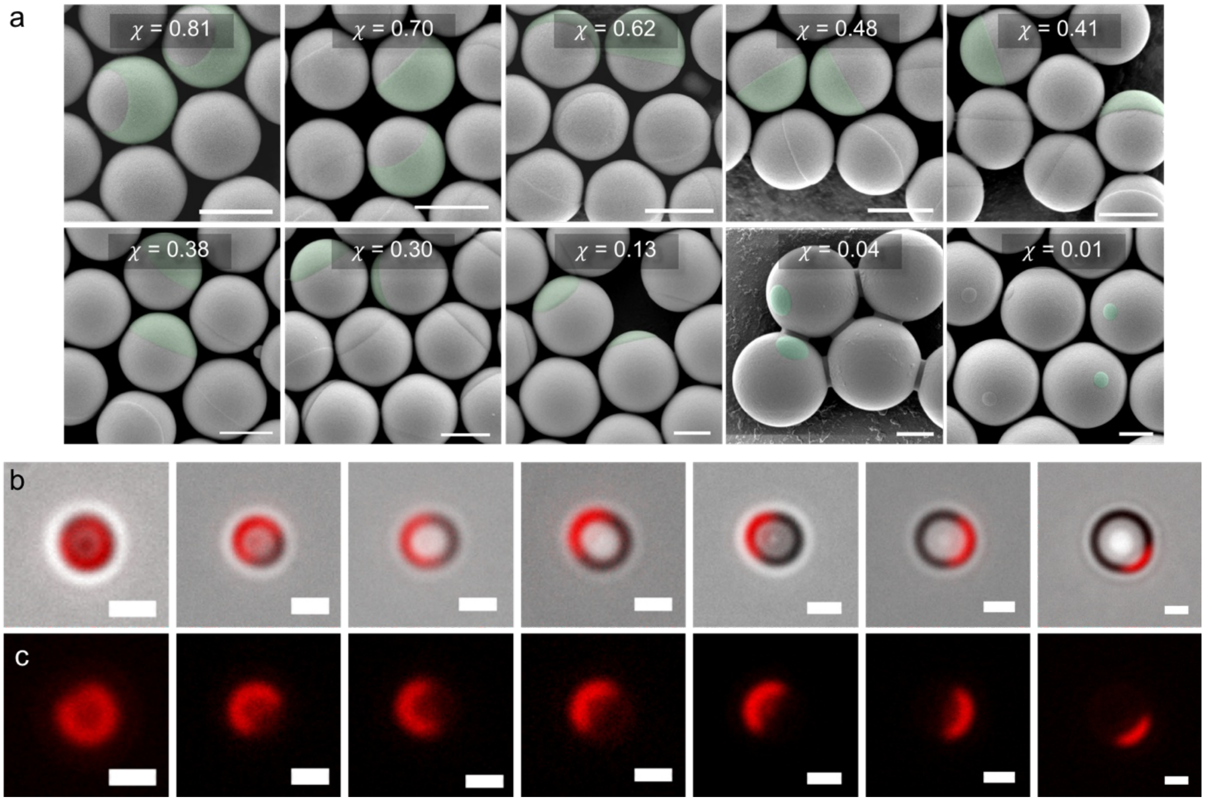

Supplementary Figure 1. PS-TPM particles and DNA-coated PS-TPM particles. a, SEM images show PS-TPM patch particles with various patch ratios, where the colored region represents the PS patch. **b,** Bright-field images of DNA-coated PS-TPM particles (overlaid with fluorescent images in **c** of DNA-coated PS-TPM particles with various patch ratios (0.81, 0.62, 0.48, 0.41, 0.38, 0.3, and 0.13). Scale bars: 1 μm.

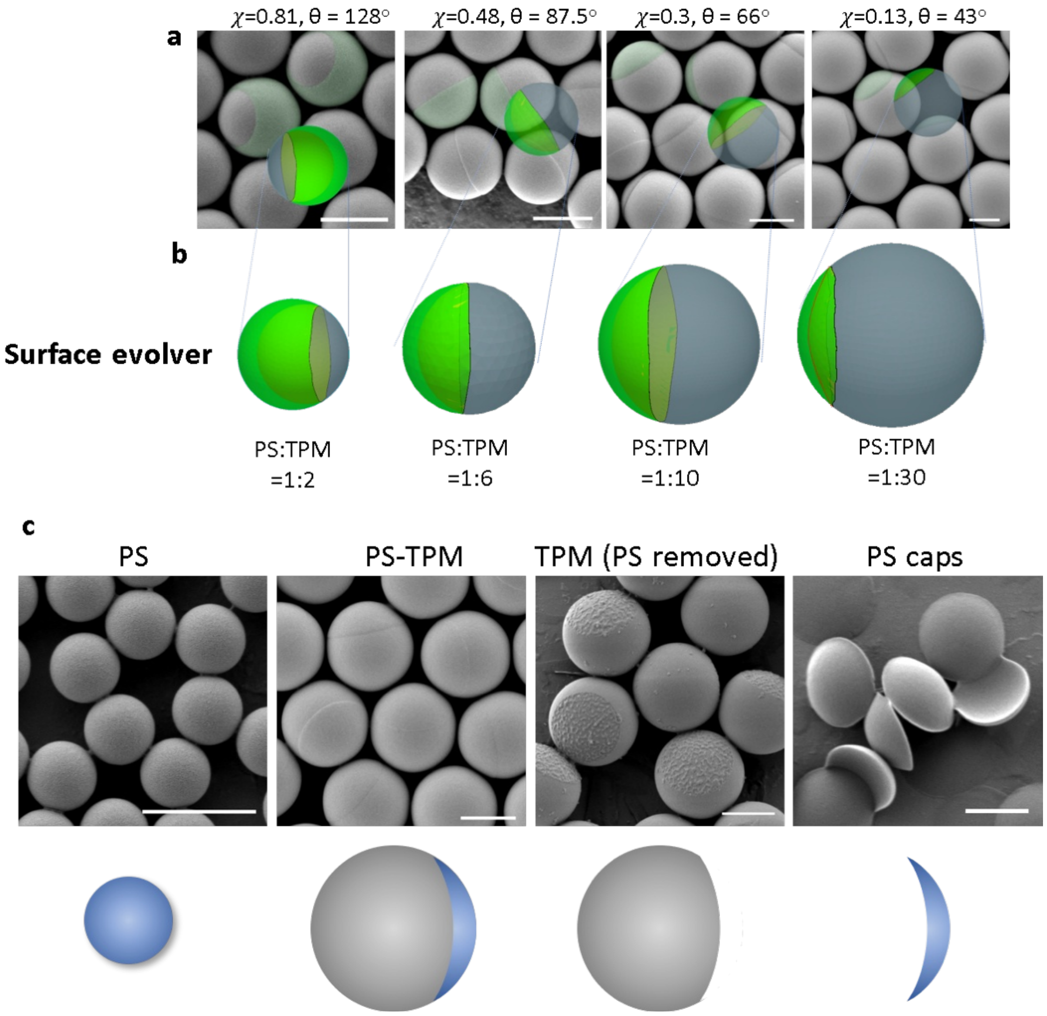

Supplementary Figure 2. PS-TPM particles. a, SEM images of PS-TPM particles. **b,** Simulated particles using a 'Surface Evolver' simulation. **c,** SEM images of PS particles, PS-TPM particles, TPM particles after removal of PS caps, and PS caps after removal of TPM. Scale bars 1 μm.

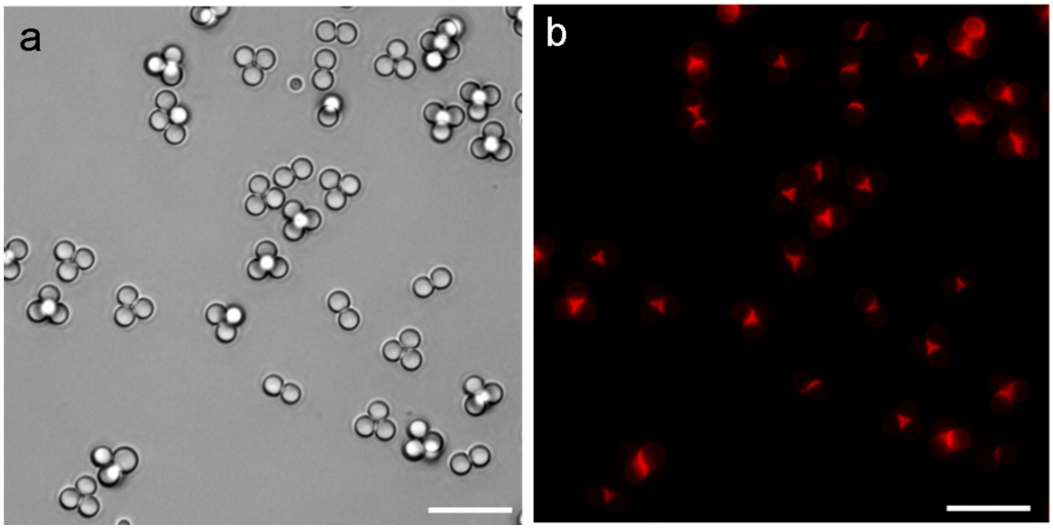

Supplementary Figure 3. Self-assembly of self-complementary DNA-coated PS-TPM particles with a patch ratio of 0.13. a, b, Bright-field and fluorescent images show the self-assembled clusters of dimers, trimer, and tetramers. Red fluorescently-labeled DNA confirms the selective DNA coating of the PS surfaces. Scale bars: 10 µm. The fluorescent image confirms that the particles are connected to each other only through DNA-coated PS patches.

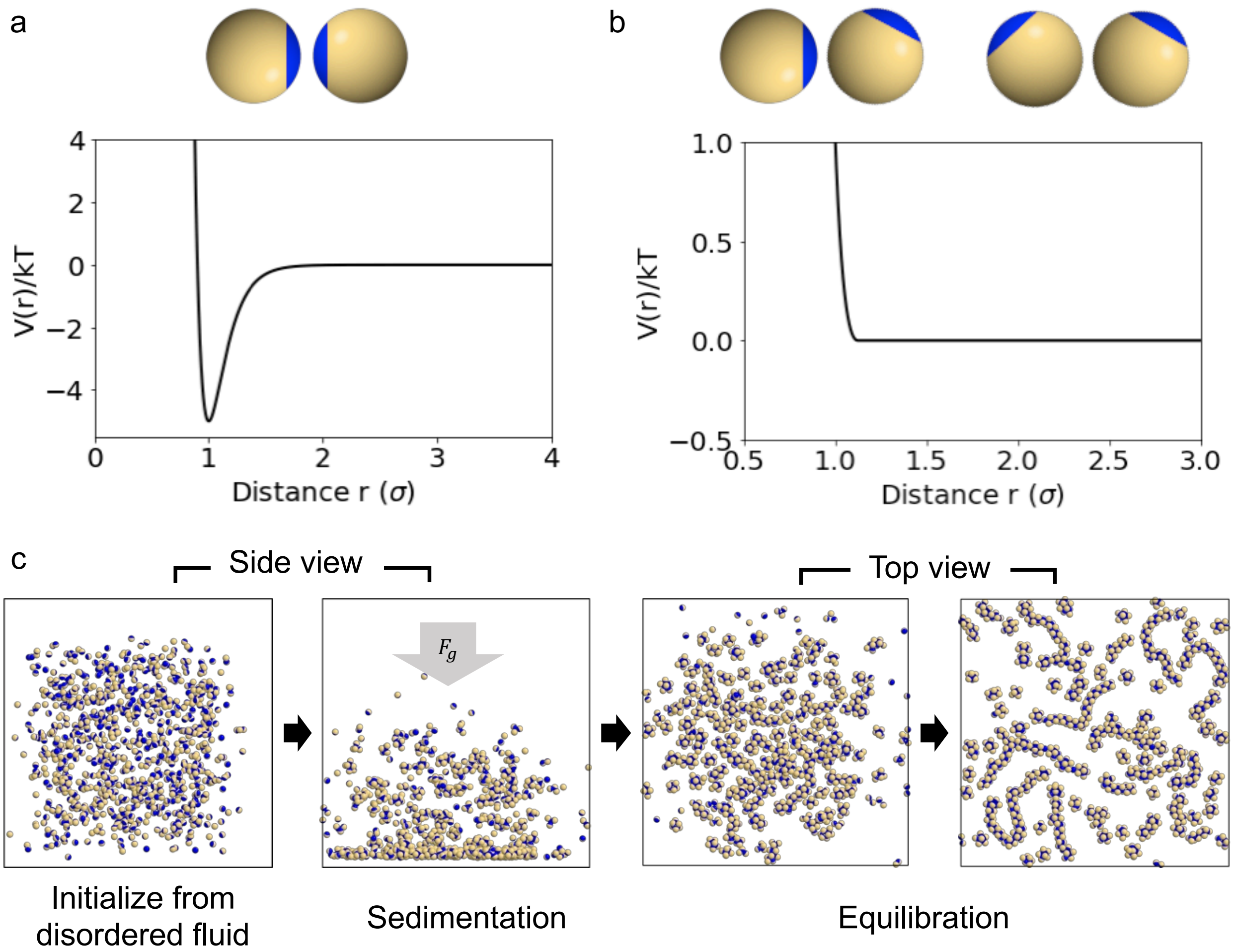

Supplementary Figure 4. Simulation model and self-assembly protocol. (a) Plot for the Morse pair potential applied between patch and patch contact at $T^* = 1.0$. (b) Plot for the WCA pair potential applied between patch and non-patch (yellow) contact and between non-patch and non-patch contact at $T^* = 1.0$. On the top of the plots, examples of each contact are shown, where the blue part is DNA-coated patch region and the yellow part is non-patch region. (c) Simulation protocol for self-assembly of N=1,000 Janus particles with $\chi = 0.35$ at $T^* = 0.7$. From left to right, the snapshots are captured at $10^5\tau$, $5 \times 10^5\tau$, $10^6\tau$ and $10^7\tau$ simulation time steps.

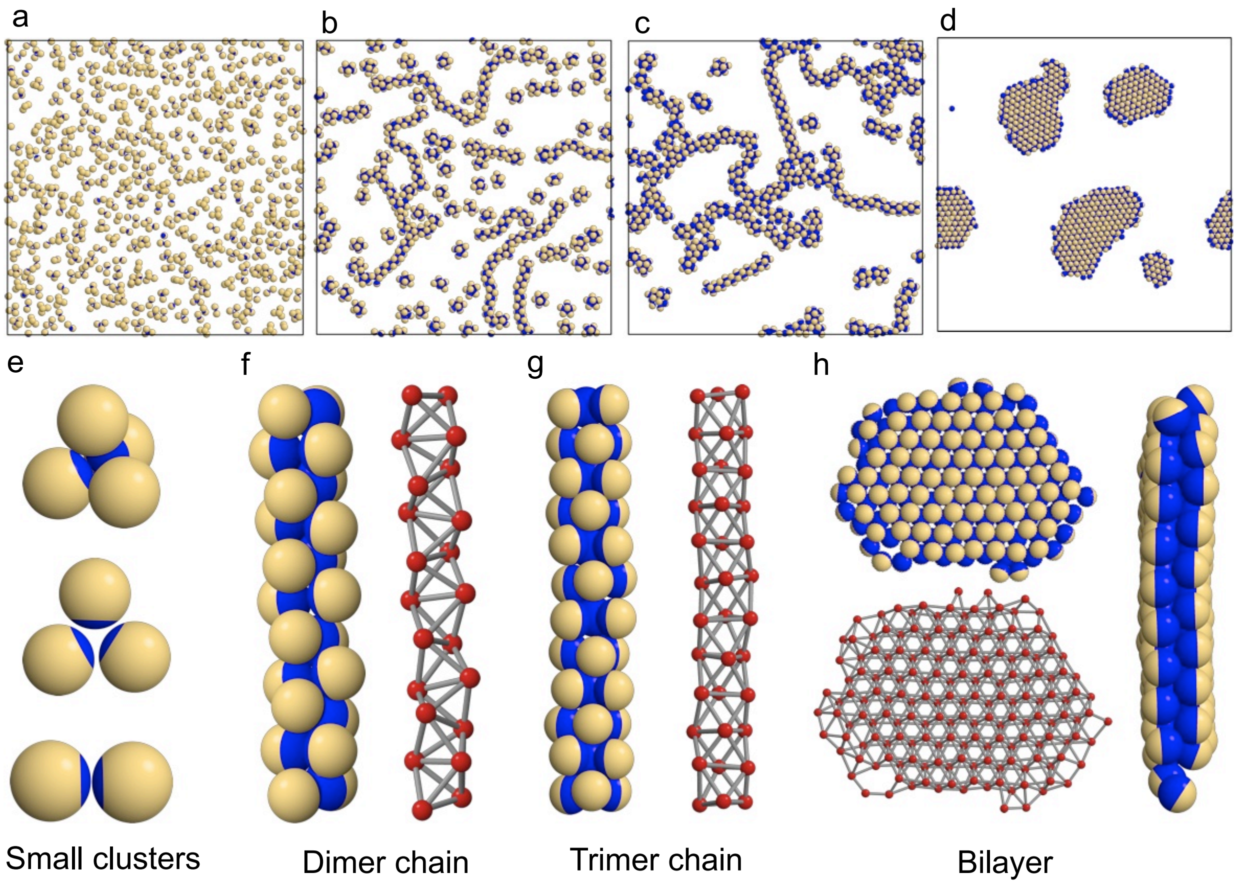

Supplementary Figure 5. Self-assembly results of the MD simulation. (**a-d**) Simulation snapshots ($\tau = 10^8$) of N=1,000 Janus particles with **a**, $\chi = 0.13, \mathrm{T}^* = 0.40$, **b**, $\chi = 0.35, \mathrm{T}^* = 0.70$, **c**, $\chi = 0.475, \mathrm{T}^* = 0.90$ and **d**, $\chi = 0.6, \mathrm{T}^* = 1.25$. Depletion effect is not considred. (e) Small clusters found in $\chi = 0.13$ system. Tetramer (top), trimer (middle) and dimer (bottom). (f) Dimer chain shown in Janus particle representation (left) and ball-and-sticks representations (right) found in $\chi = 0.35$ system. The ball-and-sticks image shows centers of the Janus particles (red) and bonds (grey) with nearest neighbors. (g) Trimer chain shown in Janus particle representation (left) and ball-and-sticks representations (right) found in $\chi = 0.475$ system. (h) Top view (left top and bottom) and side view (right) of bilayer found in $\chi = 0.60$ system.

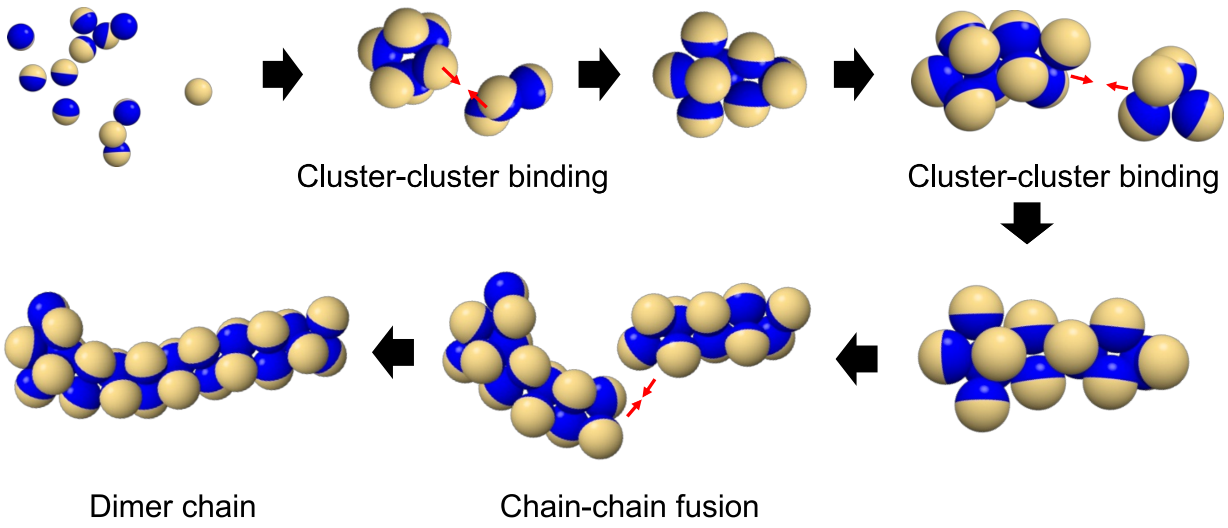

Supplementary Figure 6. Dimer chain formation in the MD simulation. Tracking the formation process of a dimer chain in $\chi = 0.35$ system at $T^* = 0.55$. A dimer chain grows via the cluster-cluster binding and the chain-chain fusion. See Supplementary Movie 3 for the complete process of the formation.

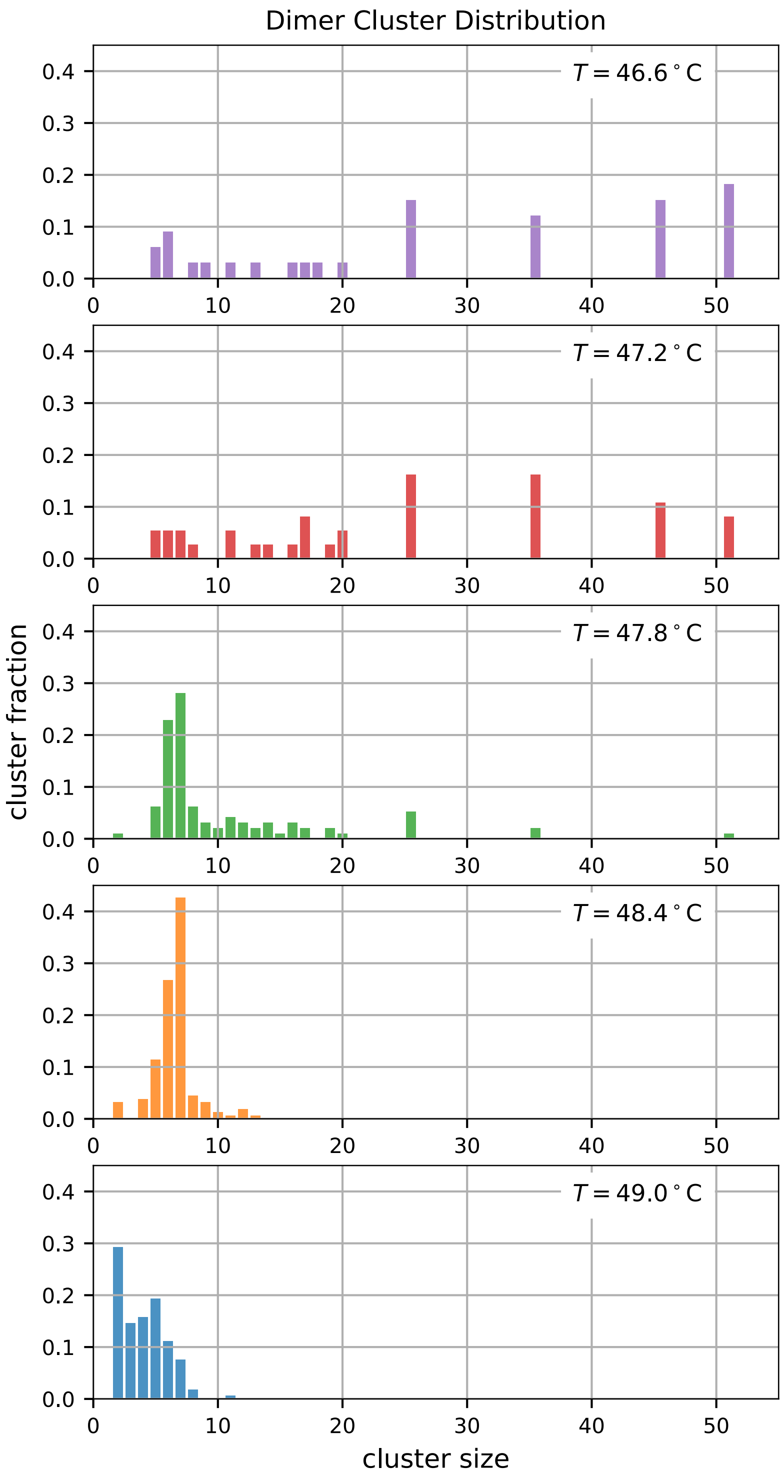

Supplementary Figure 7. Fraction of clusters in clusters of different sizes as a function of temperature for a patch ratio of 0.3. At higher temperatures, small clusters with 8 or fewer particles dominate. As the temperature is lowered, chains develop and grow longer, mostly by the addition of small clusters but occasionally by fusion of long chains. For cluster sizes greater than 20, the histogram bars integrate over ten or more bins: 21-30, 31-40, 41-50, and >50.

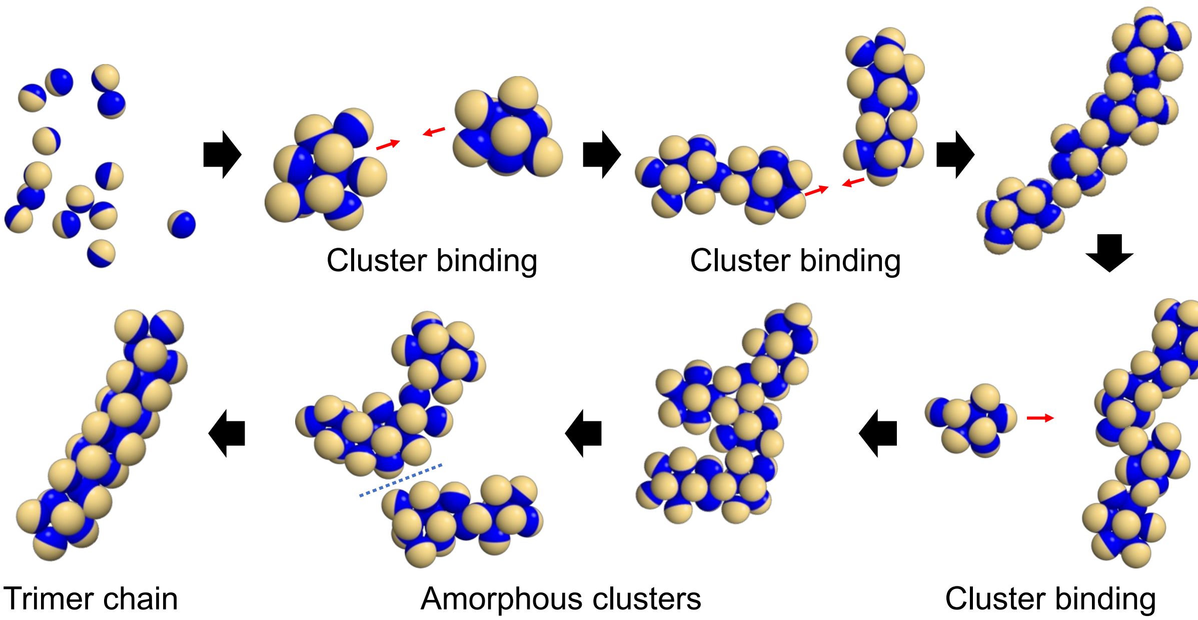

Supplementary Figure 8. Trimer chain formation in the MD simulation. Tracking the formation process of a trimer chain in $\chi = 0.475$ system at $T^* = 0.8$. Isotropic fluid forms amorphous chains (or clusters) first via the cluster binding, and they condense into ordered trimer chains. See Supplementary Movie 6 for the complete process of the formation.

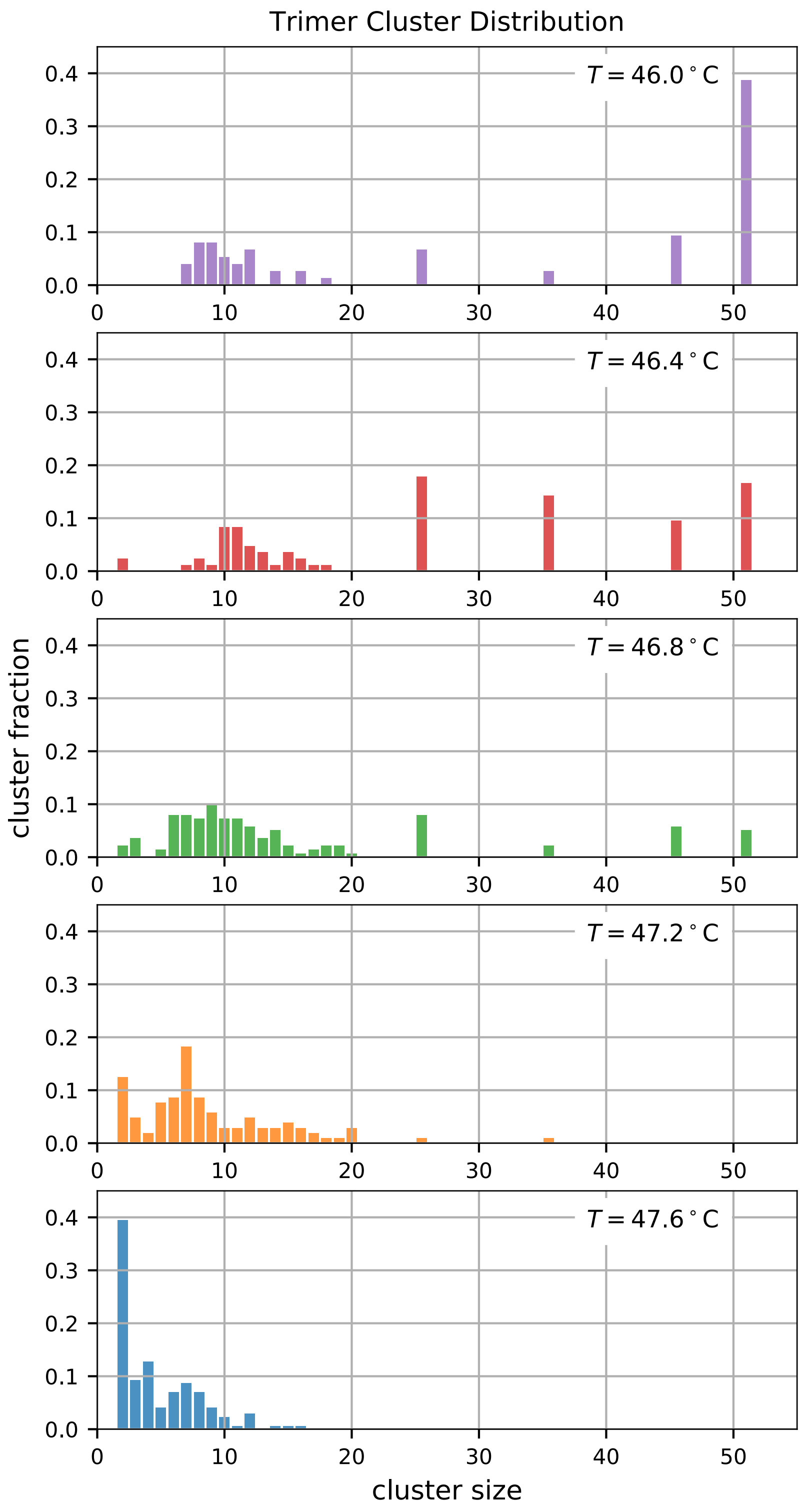

Supplementary Figure 9. Fraction of clusters that are found in clusters of different size as a function of temperature for a patch ratio of 0.48. For cluster sizes greater than 20, the histogram bars integrate over ten or more bins: 21-30, 31-40, 41-50, >50.

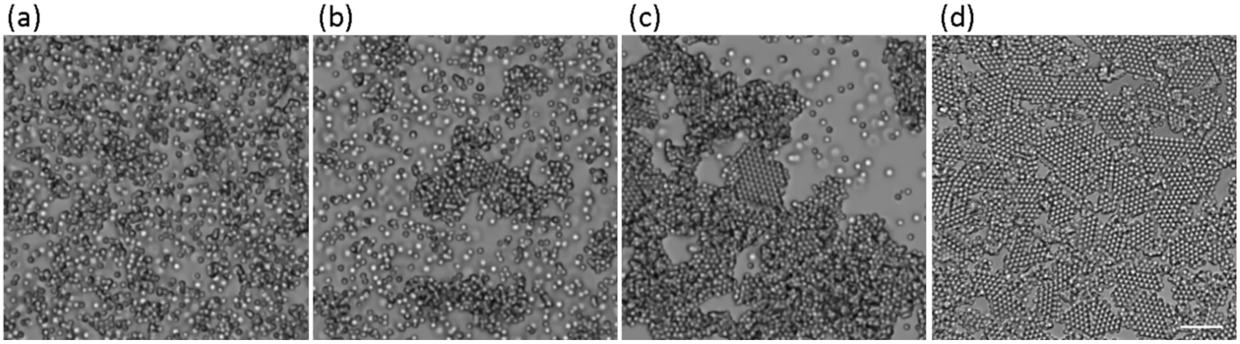

Supplementary Figure 10. Self-assembly of patch particles with a patch ratio of 0.6 into bilayer structures (stills from Supplementary Movie 7). (a) patch particles start to bind in the beginning of annealing, forming disordered aggregates, (b and c) the structure of aggregates gradually arranges into hexagonally-packed bilayers, and (d) particles grow on the edge of bilayers to form larger bilayers. Scale bar 10 μm

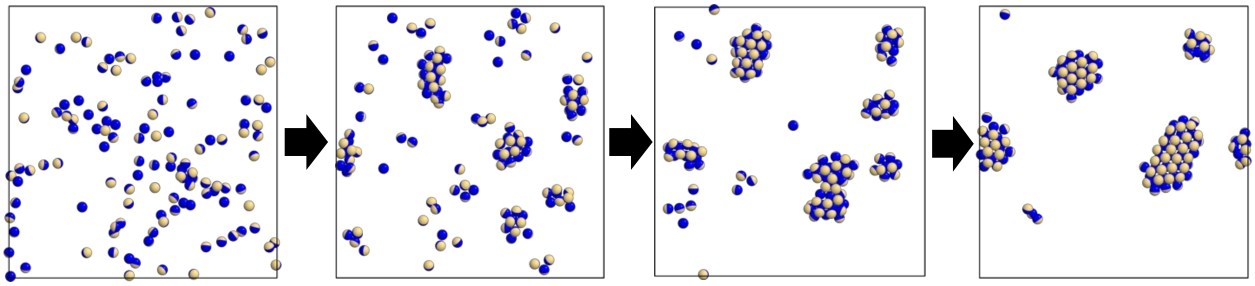

Supplementary Figure 11. Bilayer formation in the MD simulation. Tracking the formation process of bilayers in $\chi = 0.60$ system at $T^* = 1.25$. A fluid of Janus particles forms small amorphous clusters which aggregate into larger clusters. Then, they gradually rearrange into hexagonally-packed bilayers. See Supplementary Movie 8 for the complete process of the formation.

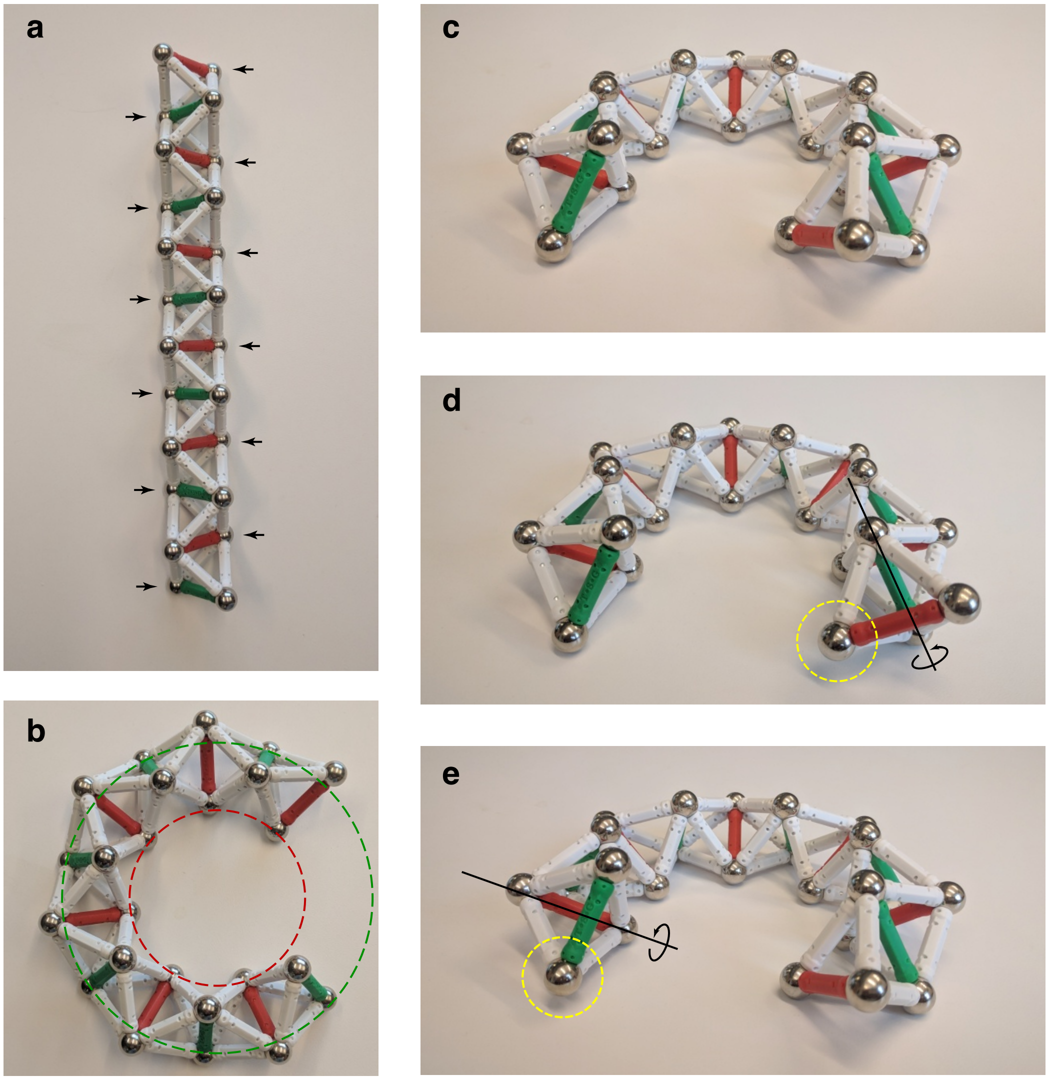

Supplementary Figure 12. Half of the particles can touch the substrate only if the radius of curvature of the chain is constant. Dimer chains are modeled with a magnetic toy. (a) For a straight chain, exactly half of the particles can touch the substrate, as indicated by the arrows. (b-c) A chain bends by rotating about axes defined by the red and green bars. Half of the particles can touch the substrate, shown by the red and green dashed circles, if the radius of curvature is constant. (d) Rotating about the last green axis causes the last particle, circled by a yellow dashed line, to lift off of the substrate, which must happen for any deviation from constant chain curvature. (e) Similarly, rotating about the last red axis causes the last particle to lift off of the substrate, which must happen for any deviation from constant chain curvature. Successive application of this condition down the chain enforces the constant curvature condition.

.

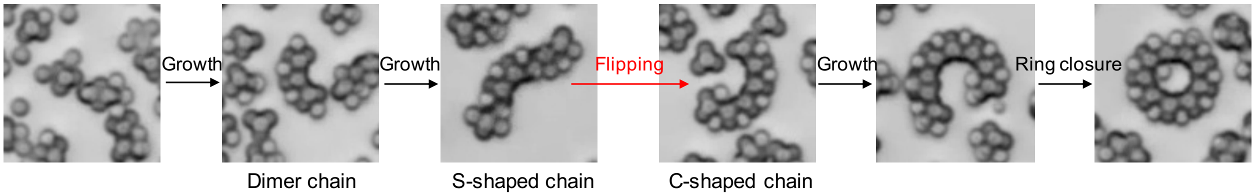

Supplementary Figure 13. Optical images show the formation of a ring. Formation of a dimer chain by cluster-cluster addition, transition from a S-shaped chain to a C-shaped chain by flipping, chain growth, and a ring-closure (stills from Supplementary Movies 9 and 10).

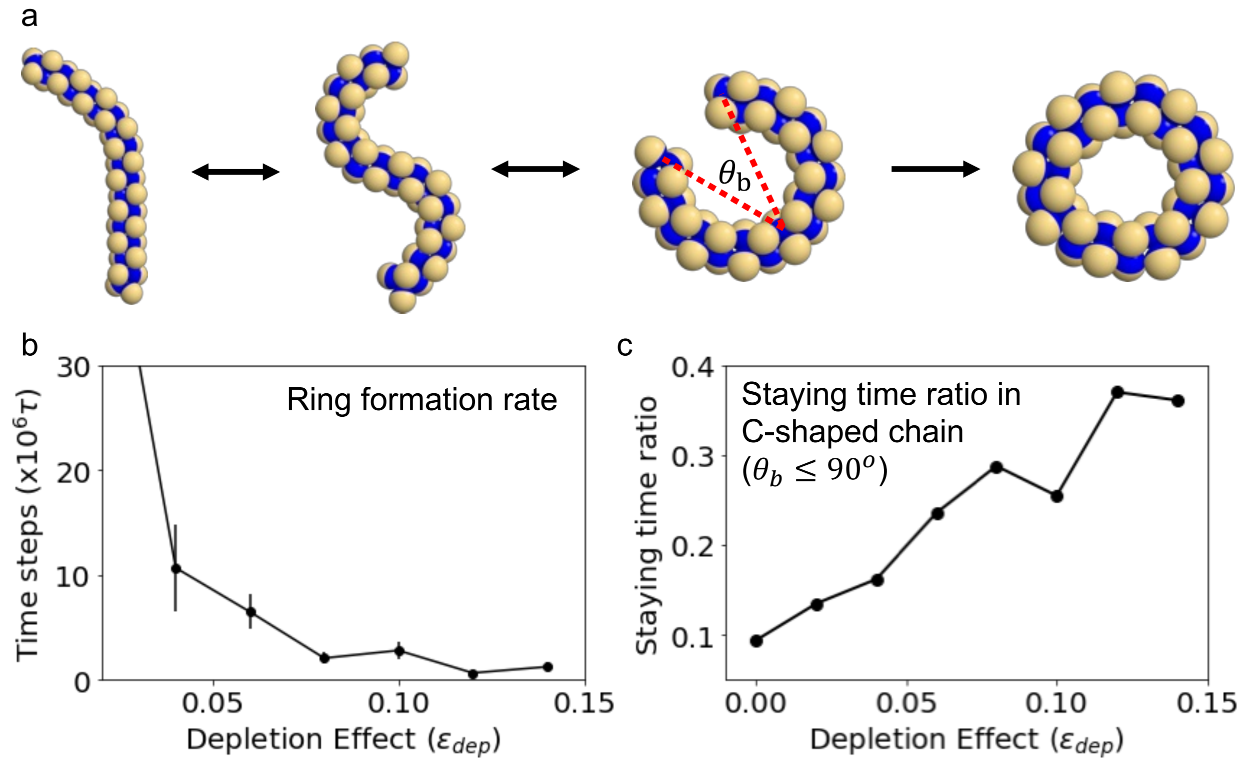

Supplementary Figure 14. **Ring formation in the MD simulation.** (a) Tracking the formation process of ring in $\chi = 0.35$ system at $T^* = 0.55$ with $\varepsilon_{\text{dep}} = 0.1$ depletion effect, initialized from a flexible dimer chain. The dimer chain fluctuates between S-shaped and C-shaped chain and eventually forms a closed ring chain. (b) Ring formation rate depends on the strength of depletion effect (ε_{dep}), averaged by 10 different runs. As the depletion effect increases, the ring formation rate becomes faster. When there is no depletion effect, ring formation was not observed within the simulation time ($\tau = 10^8$). (c) The ratio of staying time in C-shaped chain before ring-formation, averaged by the 10 different runs. When the bent angle (θ_b) of the chain (third image in **a**) is less then 90°, we considered the chain is in C-shape. As depletion effect increases, the staying time ratio in the C-shape increases.

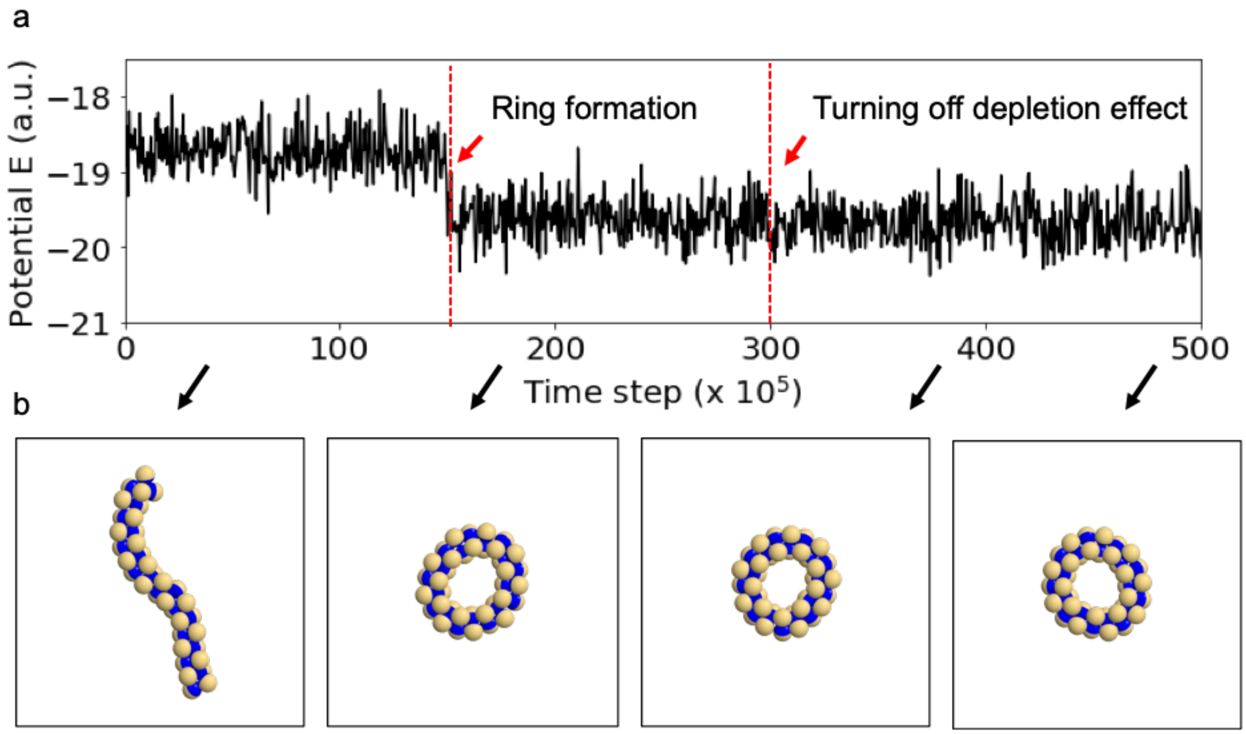

Supplementary Figure 15. Potential energy plot for ring formation. (a) Time evolution of DNA-patch potential energy (V_{P}) per particle in the presence of depletion effect ($0 \leq \tau \leq 3 \times 10^7$, $\varepsilon_{dep} = 0.1$) and in the absence of the depletion effect ($3 \times 10^7 < \tau$, $\varepsilon_{dep} = 0.0$) at constant temperature ($\mathrm{T}^* = 0.55$). A dimer chain forms a ring, resulting in a sudden energy drop around $\tau = 1.5 \times 10^7$. The ring structure is stable after turning off the depletion effect ($\tau = 3 \times 10^7$), indicating that the ring is energetically more stable than the dimer chain. (b) Simulation snapshots at 0.5×10^7, 2.0×10^7, 4.0×10^7 and 5.0×10^7 time step τ.

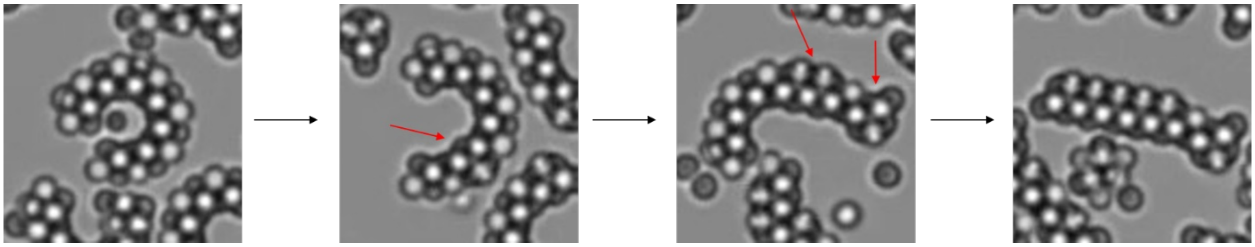

Supplementary Figure 16. Reconfiguration of a dimer chain into a compact chain through local rearrangements. Red arrows point the octahedral defects.

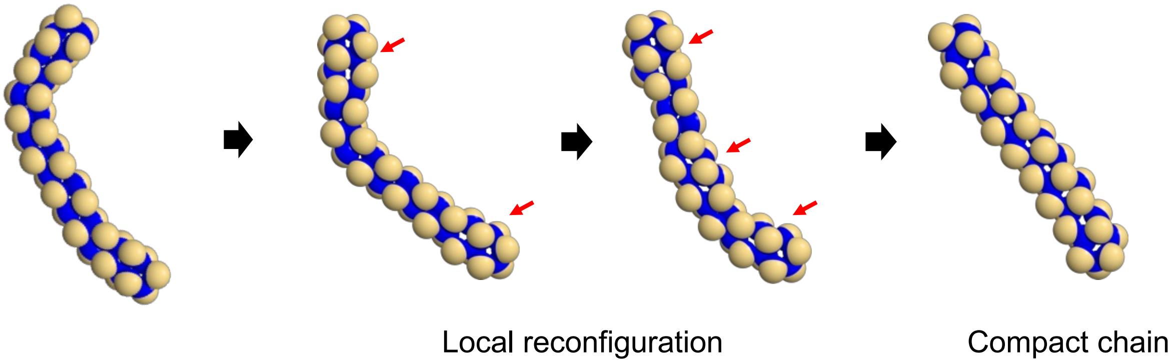

Supplementary Figure 17. Compact chain formation in the MD simulation Tracking the formation process of a compact chain in $\chi = 0.425$ system at $T^* = 0.5$ with $\varepsilon_{dep} = 0.1$ depletion effect, initialized from a flexible dimer chain. The dimer chain shows local reconfiguration into a more compact and non-flexible structure that expands to the whole chain at the end.
