# Supplementary material for: Colloidal fibers and rings by cooperative assembly
Source: Nat Commun. 2019 Sep 2;10:3936. doi: 10.1038/s41467-019-11915-1 (PMC6718632; doi:10.1038/s41467-019-11915-1)
Supplement: Supplementary file 2 — Description of Additional Supplementary Files [file 41467_2019_11915_MOESM2_ESM.pdf]

Supplementary Movie Captions

Supplementary Movie 1.
Description: Thermally reversible assembly of DNA-coated patchy particles (χ = 0.34). 100 times faster than actual time.

Supplementary Movie 2. Formation of chain structures.
Description: DNA-coated patchy particles (χ = 0.34). 300 times faster than actual time.

Supplementary Movie 3.
Description: Formation of dimer chains (simulation).

Supplementary Movie 4.
Description: Formation of dimer chains from singlets to clusters and to chains.

Supplementary Movie 5.
Description: Formation of trimer chain.

Supplementary Movie 6.
Description: Formation of trimer chains (simulation).

Supplementary Movie 7.
Description: Formation of bilayers (χ = 0.6). 1800 times faster than actual time.

Supplementary Movie 8.
Description: Formation of bilayers (simulation).

Supplementary Movie 9.
Description: Transition from a S-shaped chain to a C-shaped chain. 210 times faster than actual time.

Supplementary Movie 10.
Description: Formation of bent chains, rings and compact chains (χ = 0.34, F127 1%). 1500 times faster than actual time.

Supplementary Movie 11.
Description: A semiflexible compact chain with a kink. 100 times faster than actual time.
